# Are delusions and/or referentiality associated with aberrant reward prediction error (RPE) signaling? Evidence from fMRI using a probabilistic monetary reward task

**DOI:** 10.1017/S0033291724003258

**Published:** 2025-02-20

**Authors:** María Ángeles García-León, Paola Fuentes-Claramonte, Abigail Gee, Nuria Ramiro-Sousa, Joan Soler-Vidal, Pilar Salgado-Pineda, Llanos Torres, Nuria Jaurrieta, Manel Sánchez-Pérez, Francesco Panicali, Emilio J. Inarejos Clemente, Joaquim Raduà, Salvador Sarró, Raymond Salvador, Peter J. McKenna, Edith Pomarol-Clotet

**Affiliations:** 1 Department of Personality, Assessment, and Psychological Treatments, Universidad de Sevilla, Seville, Spain; 2 FIDMAG Germanes Hospitalàries Research Foundation, Barcelona, Spain; 3 CIBERSAM (Centro de Investigación Biomédica en Red de Salud Mental), Instituto de Salud Carlos III, Barcelona, Spain; 4 Cambridgeshire and Peterborough NHS Foundation Trust, Cambridge, UK; 5 University of Cambridge, Cambridge, UK; 6 Fundació Hospitalàries Barcelona - Hospital General, Barcelona, Spain; 7 Fundació Hospitalàries Sant Boi, Sant Boi de Llobregat, Barcelona, Spain; 8 Fundació Hospitalàries Barcelona, Barcelona, Spain; 9 Fundació Hospitàries Martorell, Martorell, Barcelona, Spain; 10 Universitat Autònoma de Barcelona, Barcelona, Spain; 11 Departament de diagnòstic per la Imatge, Hospital Sant Joan de Déu, Barcelona, Spain; 12 Institut d’Investigacions Biomèdiques August Pi i Sunyer (IDIBAPS), Barcelona, Spain; 13 University of Barcelona, Barcelona, Spain

**Keywords:** schizophrenia, delusions, referentiality, reward, reward prediction error, fMRI

## Abstract

**Background:**

According to the aberrant salience proposal, reward processing abnormality, specifically erroneous reward prediction error (RPE) signaling due to stimulus-independent release of dopamine, underlies delusions in schizophrenia. However, no studies to date have examined RPE-associated brain activations in relation to this symptom.

**Methods:**

Seventy-eight patients with a DSM-5 diagnosis of schizophrenia/schizoaffective disorder and 43 healthy individuals underwent fMRI while they performed a probabilistic monetary reward task designed to generate a measure of RPE. Ratings of delusions and referentiality were made in the patients.

**Results:**

Using whole-brain, voxel-based analysis, schizophrenia patients showed only minor differences in RPE-associated activation compared to healthy controls. Within the patient group, however, severity of delusions was inversely associated with RPE-associated activation in areas including the caudate nucleus, the thalamus and the left pallidum, as well as the lateral frontal cortex bilaterally, the pre- and postcentral gyrus and supplementary motor area, the middle cingulate gyrus, and parts of the temporal and parietal cortex. A broadly similar pattern of association was seen for referentiality.

**Conclusions:**

According to this study, while patients with schizophrenia as a group do not show marked alterations in RPE signaling, delusions and referentiality are associated with reduced activation in parts of the prefrontal cortex and the basal ganglia, though not specifically the ventral striatum. The direction of the changes is on the face of it contrary to that predicted by aberrant salience theory.

Delusions – false, bizarre, and sometimes fantastic beliefs that are held with fixed conviction and are not susceptible to counter-argument – are one of the defining symptoms of schizophrenia. While the cause or causes of the symptom remain uncertain, recent years have seen the emergence of a number of testable theories (for reviews see Feyaerts et al., 2021; Freeman, 2007; McKenna, 2017). These include Garety and co-workers’ (e.g., Garety & Freeman, 2013) proposal that delusions reflect a probabilistic reasoning bias (‘jumping to conclusions’) when decisions are made under conditions of uncertainty, and Frith’s (Frith, 1992) suggestion that persecutory and referential delusions are due to an acquired (as opposed to a developmental) theory of mind deficit. Findings for both these approaches have not been fully supportive, however (for a review see McKenna, 2017).

A third, highly influential, approach to delusions is the ‘aberrant salience’ theory of Heinz (2002) and Kapur (2003). Drawing on the dopamine hypothesis of schizophrenia (e.g., Howes & Kapur, 2009) and the demonstration by Schultz et al. (1997) that dopamine codes a reward prediction error (RPE) signal in the brain, this proposal argues that increased dopamine transmission in schizophrenia will lead to non-stimulus-dependent release of dopamine, causing motivational and reinforcing properties – that is, salience – to become attached to neutral stimuli in the environment. This would then result in a state where environmental events spuriously acquire significance, something that is similar if not identical to the symptom of delusional mood.

The aberrant salience theory is readily extendable to other classes of delusion where abnormal significance is a feature, that is, delusions of reference and misinterpretation, but how aberrant salience might lead to the full range of delusions seen in schizophrenia was not addressed in Heinz’s (2002) and Kapur’s (2003) accounts, beyond suggestions that the patient’s attempts to make sense of the experience of aberrant salience might be important. Subsequently, however, Fletcher and Frith (2009) and other authors (Adams et al., 2013; Corlett et al., 2009; Corlett et al., 2010) have extended the aberrant salience theory to explain how non-significance-bearing delusions might develop. They point out that prediction errors are formed when existing predictive models about the world are violated, and that these prediction errors will in turn modify predictive models. When aberrant salience causes reward prediction errors to be generated erroneously, erroneous predictive models will therefore also be generated; however, these can never be successful because they can never eliminate the prediction error, leading the patient’s explanatory models of the world to deviate further and further from reality (for a review see McKenna, 2024).

Functional imaging studies during performance of monetary and other reward tasks have provided clear evidence that reward processing is altered in schizophrenia. Specifically, viewing of stimuli that are predictive of reward (‘reward anticipation’) is associated with reduced activation in the ventral striatum, as well as in other parts of the basal ganglia, the amygdala, the insula, the anterior and mid-cingulate cortex, the right precentral gyrus, and the right superior temporal gyrus (for meta-analyses, see Radua et al., 2015, Zeng et al., 2022). In contrast, receipt of information that reward has been won (‘reward feedback’ or ‘reward delivery’) is associated with a pattern of increased activation in schizophrenia, seen in the striatum, the amygdala and the hippocampus/hippocampal gyrus, although there is also evidence for reduced activation in the medial frontal and dorsolateral frontal cortex (Zeng et al., 2022). Meta-analyses of studies of RPE signaling itself have had conflicting findings. One, pooling data from 10 studies, found no clusters of significant difference in patients with schizophrenia compared to healthy controls (Yaple et al., 2021). However, another, carried out on a somewhat different set of 14 studies (Yang et al., 2024), found increased activation in the left postcentral gyrus and right middle frontal gyrus in schizophrenia and decreased activation in the striatum, anterior cingulate cortex and several other areas.

The relationship of altered reward processing to the symptoms of schizophrenia has also been examined. There is meta-analytic support for an inverse association between negative symptoms and reward anticipation-related activations in the ventral striatum (Radua et al., 2015; Zeng et al., 2022), and between positive symptoms and decreased reward feedback-associated activation in the left medial frontal cortex (Zeng et al., 2022). With respect to RPE, an inverse association with negative symptoms was found in the ventral striatum in one study (Katthagen et al., 2020) but not, or only equivocally, in two others (Culbreth et al., 2016; Fuentes-Claramonte et al., 2023); one of these studies instead found reduced activation in the lateral prefrontal cortex associated with negative symptoms (Fuentes-Claramonte et al., 2023). A small number of studies examining the relationship between RPE-associated activations and positive symptoms have not had convergent findings (Culbreth et al., 2016; Gradin et al., 2011; Katthagen et al., 2020; Murray et al., 2008).

Despite being a key prediction of the aberrant salience theory, no studies to date have examined the association between reward processing and delusions in schizophrenia. In the present study, we examined this in a large group of patients with schizophrenia, both in relation to delusions and to the experience of referentiality, something that is central to the aberrant salience theory. We focused specifically on RPE, on the basis that this is the aspect of reward processing that is most closely linked to dopamine function and aberrant salience at the theoretical level.

## Methods

### Participants

The patient sample consisted of 78 patients with a DSM-5 diagnosis of schizophrenia or schizoaffective disorder, made on the basis of clinical interview and review of case notes. They were selected from a larger group of 103 patients recruited from four different hospitals in the Barcelona area (Fundació Hospitalàries Sant Boi, Fundació Hospitalàries Barcelona - Hospital General, Fundació Hospitalàries Martorell, Fundació Hospitalàries Barcelona). Patients were excluded if they (a) were younger than 18 or older than 65 years, (b) had a history of brain trauma or neurological disease, or (c) had shown alcohol/substance abuse within 12 months prior to participation. They were also required to have a premorbid IQ in the normal range (≥70), as estimated using the Word Accentuation Test (Test de Acentuación de Palabras, TAP; (Del Ser et al., 1997, Gomar et al., 2011), which requires pronunciation of low-frequency Spanish words whose accents have been removed. Patients with a current IQ of <70, based on four subtests from the WAIS-III (Vocabulary, Similarities, Matrix Reasoning and Block Design), were also excluded. All patients were right-handed and were taking antipsychotic medication.

The control sample consisted of 43 right-handed healthy individuals recruited from non-medical hospital staff, their relatives, and acquaintances, plus independent sources in the community. They were selected from a larger cohort of healthy subjects to be similar to the patients in terms of age, sex, and TAP-estimated IQ. The controls met the same exclusion criteria as the patients. They were also excluded if they reported a history of mental illness, based on the Structured Clinical Interview for DSM-IV (First et al., 2002) (modified to make it compatible with DSM-5), or were on treatment with psychotropic medication, or had a history of major mental illness in a first-degree relative.

All participants gave written informed consent prior to participation. The study was approved by the ethics committee of the Hermanas Hospitalarias group of hospitals (Comité de Ética e Investigación Clínica de Hermanas Hospitalarias). Healthy controls received a gift card as a compensation for their participation.

### Clinical measures

Symptoms were evaluated using the Positive and Negative Syndrome Scale (PANSS) (Kay et al., 1987), with positive (i.e., reality distortion, delusions and hallucinations), negative and disorganization syndrome scores being calculated based on Wallwork et al. (2012). Delusions were rated using the relevant subscale of the Psychotic Symptoms Rating Scale (PSYRATS) (Haddock et al., 1999). Referentiality was evaluated using the Ideas of Reference Interview Scale (IRIS) (Wong et al., 2012). This rates referential experiences of multiple types, including being looked at, gossiped about and laughed at, being deliberately approached or avoided, experiencing double meanings in speech and being targeted by special messages. An initial score, pervasiveness, rates the degree of presence of self-referential ideas in the patient’s daily life. Also scored are discrepancy (a measure reflecting the specificity of self-referential experiences, that is, how likely the patient considers the experience to be directed specifically towards him/her), conviction (the degree to which the subject maintains their interpretation), and frequency during the last month. We used pervasiveness as the measure in the analyses.

### Task description

**Figure 1.**
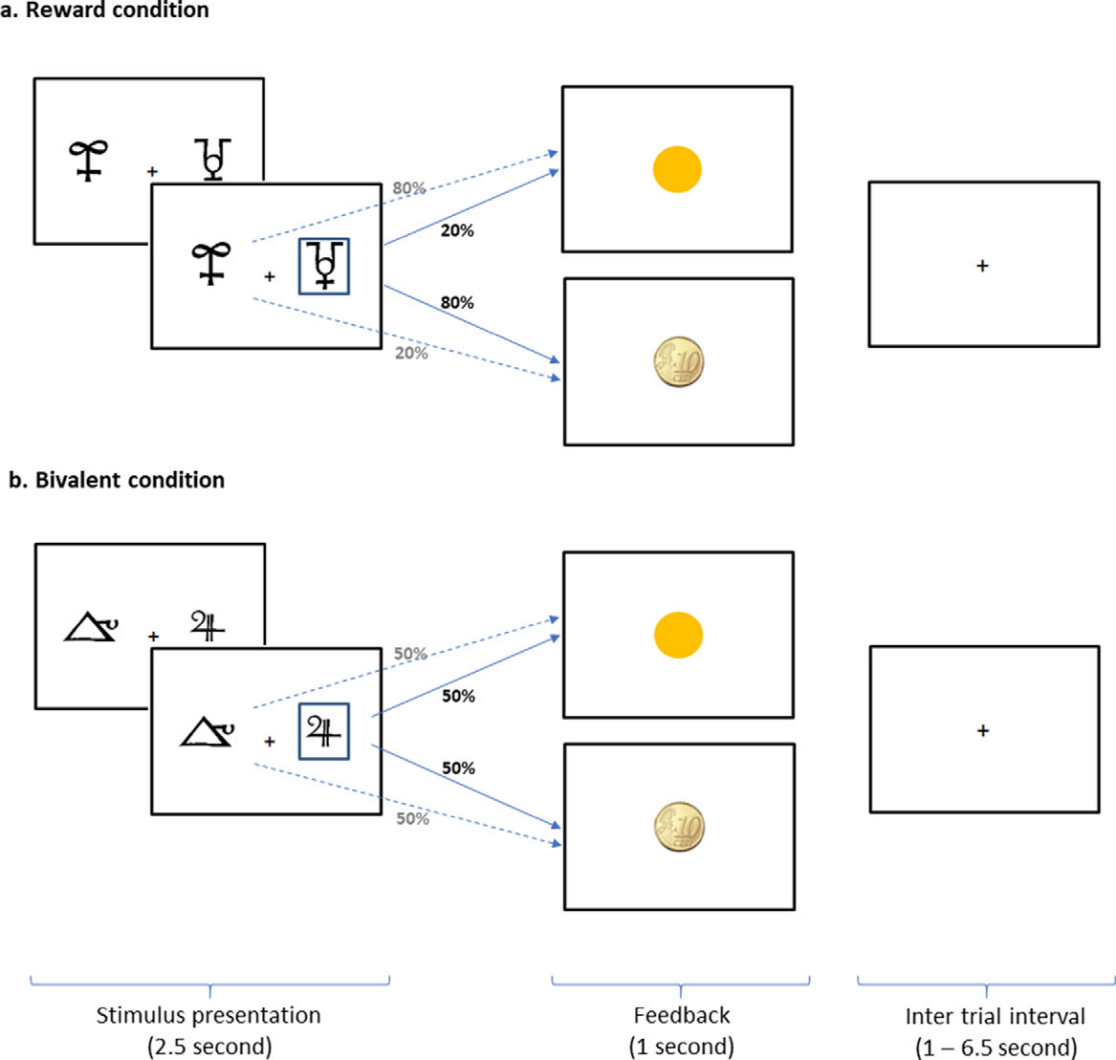
The probabilistic monetary learning task. In the reward condition, (a) one of the stimuli leads to the winning outcome (a 10-cent coin) in 80% of trials; in the bivalent condition, (b) both stimuli lead to the winning outcome in 50% of the trials.

While being scanned, subjects performed a probabilistic monetary learning task adapted from Pessiglione et al. (2006). In each trial, two stimuli were shown side by side for 2.5 s. The participant had to select one of them by pressing a button on the corresponding side. The selected stimulus was then highlighted and feedback was displayed in the form of either a 10-cent coin (win) or a yellow circle (no win) (see Figure 1). After a further 1 s, a fixation cross appeared with variable duration (range 1.0–6.5 s, mean 2.0 s) before the next trial began. Two types of stimuli were presented. In rewarded pairs, one stimulus had a winning outcome in 80% of trials, whereas the other stimulus was only rewarded on 20% of trials. In bivalent pairs, both stimuli had a winning outcome in 50% of trials. The same pair of stimuli were presented for 16 successive trials, during which (in the case of the rewarded pairs) the participant had the opportunity to learn through trial and error which of the two stimuli was associated with a winning outcome. After 16 trials, the stimuli changed and new reward contingencies had to be learned.

The task included ten different pairs of stimuli (i.e. 160 trials), half of them corresponding to rewarded pairs (1^st^, 3^rd^, 4^th^, 5^th^ and 7^th^) and the other half to bivalent pairs. The subjects were informed that the money they won would be given to them after the scanning session.

Participants underwent 48 practice trials before the scanning session. While being scanned, to ensure that they learned during the task, we excluded subjects who chose the optimal stimulus in less than 50% of the trials during the last eight trials of each rewarded pair (indicating they had not learnt to choose the rewarded stimulus after 16 trials). Subjects who showed no response or anticipated responses (reaction times <100ms) in more than 10% of the trials were also excluded.

### Computational model

RPE, defined as the difference between the expected and the actual outcome, was estimated using the Q-learning model (Watkins & Dayan, 1992). For each pair of stimuli, the model estimates the expected value of choosing stimulus A (EV_A_) and stimulus B (EV_B_) based on the subject’s sequence of choices. Expected values were set to zero at the beginning of each 16-trial block, and the expected value of the chosen stimulus was updated after each trial as a function of RPE:

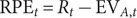




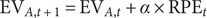

where RPE*_t_* is the reward prediction error at time *t* (the trial that has just finished), R*_t_* is the reward at time *t*, EV_*A*,*t*_ is the expected value of *A* (the chosen stimulus) at time *t*, EV_*A*,*t*+1_ is the expected value of *A* at time *t* + 1 (the following trial), and the parameter *α* is the rate of learning for the chosen stimulus. This parameter, which ranges between zero and one, weighs the influence of RPE on the updating of the expected value. A high *α* value indicates a strong influence of recent outcomes, whereas a low *α* value indicates slow learning.

The model also estimates the trial-by-trial probability of choosing each stimulus based on the expected values using a softmax function (Gläscher & O’Doherty, 2010). As an example, the probability of choosing stimulus A:

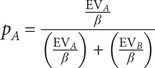

where *β* is the (inverse) temperature, a parameter that adjusts the trade-off between exploration and exploitation, and reflects *the choice between* deterministic versus exploratory responding during the task. The higher the *β* value, the more exploratory the way of choosing; the lower the *β* value, the more deterministic.

We fitted the model based on the subjects’ sequence of choices. The free parameters of the model, *α* and *β*, were estimated for each subject through the maximum likelihood technique to maximize the probability of the actual choices. For the behavioral analysis, individual parameter estimates were compared between groups using the non-parametric Kruskal–Wallis test, due to non-normal distribution of the data. For the imaging analysis, the learning model was calculated using the same parameters across subjects by using the median of the individual fitted parameters (Daw, 2011). We conducted all analyses in R 3.4.4 (R Core Team, 2022) using a softmax function.

### Image acquisition

Images were acquired with a 3T Philips Ingenia scanner (Philips Medical Systems, Best, The Netherlands). Functional data were acquired using a T2*-weighted echo-planar imaging (EPI) sequence with 443 volumes and the following acquisition parameters: TR = 2000 ms, TE = 30 ms, flip angle = 70°, in-plane resolution= 3.5 × 3.5 mm, FOV = 238 × 245 mm, slice thickness = 3.5 mm, and inter-slice gap = 0.75 mm. Slices (32 per volume) were acquired with an interleaved order parallel to the AC–PC plane. In addition, a high-resolution anatomical volume was acquired using an FFE (Fast Field Echo) sequence for anatomical reference and inspection (TR = 9.90 ms; TE = 4.60 ms; Flip angle = 8°; voxel size = 1 × 1 mm; slice thickness = 1 mm; slice number = 180; FOV = 240 mm).

Pre-processing was carried out with the FEAT module included in the FSL (FMRIB Software Library) software (Smith et al., 2004). The first six seconds (three volumes) of the sequence, corresponding to signal stabilization, were discarded. Pre-processing included motion correction (using the MCFLIRT algorithm), co-registration, and normalization to a common stereotactic space (MNI, Montreal Neurological Institute template). For accurate registration, a two-step process was used. First brain extraction was applied to the structural image, and the functional sequence was registered to it. Then the structural image was registered to the standard template. These two transformations were used to finally register the functional sequence to the standard space. Before group analyses, normalized images were spatially filtered with a Gaussian filter (FWHM = 5 mm). To minimize unwanted movement-related effects, individuals with an estimated maximum absolute movement >3.0 mm or an average absolute movement >0.3 mm were excluded from the study.

### Statistical analysis

This was by means of a General Linear Model (GLM) approach, also using the FEAT module in the FSL software. At the first level (within-subject), the following regressors were created: onset of stimuli as 2.5 s events in bivalent and reward pairs separately (two regressors) and onset of feedback as one-second events in bivalent and reward pairs separately (two regressors). Finally, RPE derived from the computational model was introduced as a parametric modulator at feedback onset as a one-second event. All regressors were convolved with a gamma response function. We added temporal derivates and motion parameters to the model. Our contrast of interest was the activation associated with the RPE regressor.

GLMs were fitted to generate whole brain individual activation maps for the contrast of interest and second level analyses were performed by means of mixed-effects GLMs (Beckmann et al., 2003), to obtain mean activation maps for each group. We then examined correlations between brain activations and symptom scores. In all these analyses, whole-brain voxel-wise statistical tests were carried out with a *p* < 0.05, cluster correction for multiple comparisons and a cluster-forming threshold of *z* > 3.1 (*p* < 0.001) using Gaussian Random Field correction (Worsley et al., 1996). In all analyses age, sex and premorbid IQ were included as covariates.

Given the prominent role of the nucleus accumbens (as part of the ventral striatum) and orbitofrontal cortex in reward processing, we additionally carried out a region-of-interest (ROI) analysis in these two regions. Separate right and left ROIs were anatomically defined using the Harvard-Oxford Cortical and Subcortical Atlases (https://fsl.fmrib.ox.ac.uk/fsl/fslwiki/Atlases).

## Results

Socio-demographic and clinical data for the patients and controls are shown in Table 1. From the initial sample of 103 patients, 12 were excluded because of excessive head motion during the fMRI session and 13 because of poor behavioral performance (as defined above), leaving a final sample of 78 patients. The patients were well matched with the 43 controls for sex, age and estimated premorbid IQ (Table 1). As expected, the patients had a significantly lower current IQ than the controls.

**Table 1.** Socio-demographic and clinical data for the patients and controls

	HC (*N* = 43)	SCZ (*N* = 78)	Statistic	*p*
Sex (M/F)	22/21	50/28	*χ2=* 1.93	0.16
Age	37.7 (13.05)	41.3 (11.50)	*t* = −1.57	0.12
Estimated premorbid IQ (TAP)	101.1 (8.90)	102.0 (9.08)	*t* = 1.59	0.11
Current IQ (WAIS-III)	105 (13.4)	93.0 (12.9)	*t* = 4.74	0.001
PANSS total	—	55.5 (16.0)		
Positive syndrome	—	9.79 (4.38)		
Negative syndrome	—	14.6 (7.49)		
Disorganization	—	5.87 (2.48)		
PSYRATS	—	11.7 (14.5)		
PSYRATS hallucinations	—	5.41 (10.02)		
PSYRATS delusions	—	6.25 (7.10)		
Antipsychotic treatment (CPZ eq.)	—	392.57 (311.67)		
Illness duration (years)	—	18.30 (11.33)		

### Behavioral data

**Figure 2.**
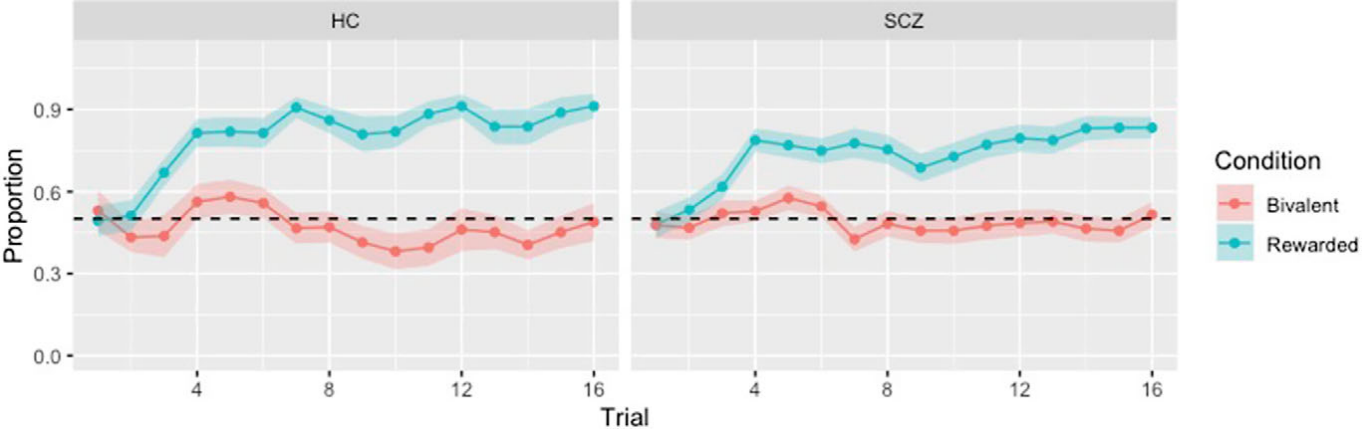
Evolution of responding throughout the task for rewarded and bivalent pairs. Shown is the proportion of trials where each group selected the rewarded stimulus in the rewarded pairs (blue) and in the bivalent pairs (red) for healthy controls and patients with schizophrenia. Shaded regions around the lines indicate 95% confidence intervals.

The proportion of correct choices was defined as that where the stimulus with the highest reward probability was chosen, irrespective of the actual outcome; in bivalent pairs, where the reward probability was equal, this measure simply reflected the proportion of choices for one of the two stimuli. The findings averaged across participants, separated for each group and condition, are shown in Figure 2, and indicate that both groups showed learning in the rewarded condition; in the bivalent condition, choice proportion remained around 50% throughout the trial sequence. Overall, the patients had a significantly lower proportion of correct choices for the rewarded pairs (*t* = 2.96; *p* = 0.004). There were no significant differences in reaction times between the patients and controls for rewarded or bivalent pairs. The two groups did not differ on the Q-learning model parameters *α* (rate of learning) (HC: 0.45 ± 0.20; SCZ: 0.39 ± 0.28; *t* = 1.12; *p* = 0.26) and *β* (temperature) (HC: 0.29 ± 0.19; SCZ: 0.30 ± 0.27; *t* = −0.15; *p* = 0.88).

Model fit, measured as the root mean square error (RMSE) of the regression between actual and predicted choices, was reduced for patients compared to controls (HC: 0.48 ± 0.02; SCZ: 0.40 ± 0.07; *t* = 7.14; *p* < 0.001). A simulation analysis of parameter recoverability was also carried out and is described in the Supplementary Material (Figure S1). It found that there was significant correlation between true and estimated parameters.

### RPE-associated brain activations in the patients and the controls

**Figure 3.**
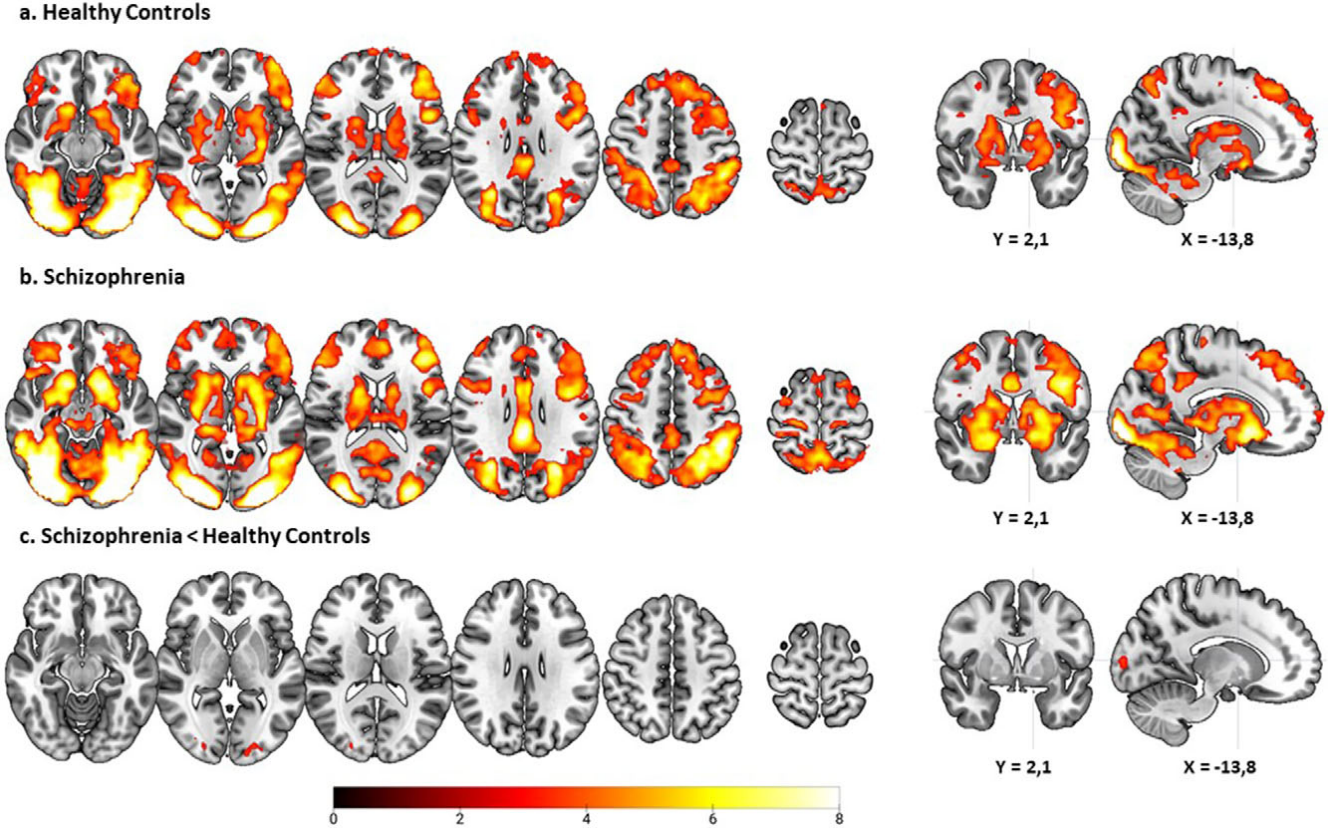
Brain activations associated with RPE in the healthy controls (a) and the schizophrenia patients (b), and the comparison control > schizophrenia (c). Images are displayed in radiological convention (left is right). The color bar depicts *z*-values.

In the healthy controls, RPE was positively associated with activation in the caudate nucleus, including its ventral sector, the putamen, and the hippocampus bilaterally. Activations were also seen in the bilateral orbitofrontal and lateral prefrontal cortex (more pronounced on the left) extending into the medial superior frontal cortex and precentral gyrus, the posterior cingulate and the bilateral inferior parietal cortex extending to visual areas (see Figure 3a). There were no clusters of negative association with RPE. (For details of co-ordinates see Supplementary Table S1).

As shown in Figure 3b, the pattern was visually similar in the patients with schizophrenia (see Figure 3b and Supplementary Table S1). Once again there were no clusters of negative association.

Group comparison (Figure 3c, Supplementary Table S1) revealed only two small clusters of significantly reduced RPE-associated activation in the patients, located in the occipital lobes (left hemisphere: cluster size (voxels) = 186, *z* = 4.36, *p* = 0.001, MNI coordinates (*x*, *y*, *z*): −22, −88, 6; right hemisphere: cluster size (voxels) = 151, *z* = 4.91, *p* = 0.004 MNI coordinates (*x*, *y*, *z*): 28, −90, 10). There were no clusters of increased RPE-associated activation.

**Figure 4.**
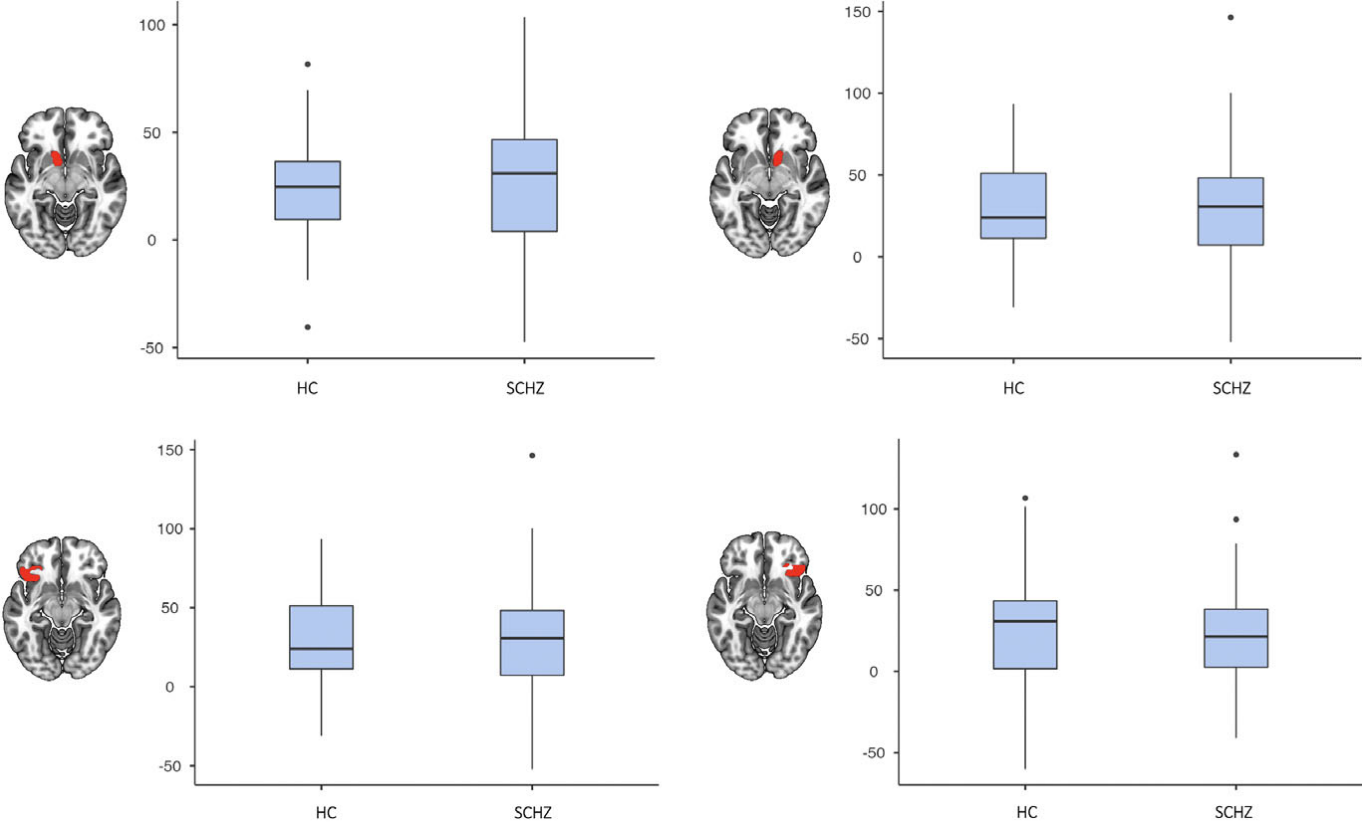
Plots for comparison of RPE-associated activation between groups in the main RPE related ROIs: accumbens (left and right on the upper row) and orbitofrontal cortex left and right on the slower row).

As shown in Figure 4, there were no significant differences in mean activation on the left or right between the schizophrenia patients and healthy controls in the anatomical ROIs in the nucleus accumbens and the orbitofrontal cortex [accumbens: right (*t* = −0.54; *p* = 0.58), left (*t* = −0.90; *p* = 0.37); orbitofrontal cortex: right (*t* = 0.52; *p* = 0.61), left (*t* = −0.54; *p* = 0.59)].

### RPE-associated activations in relation to PSYRATS delusion scores

**Figure 5.**
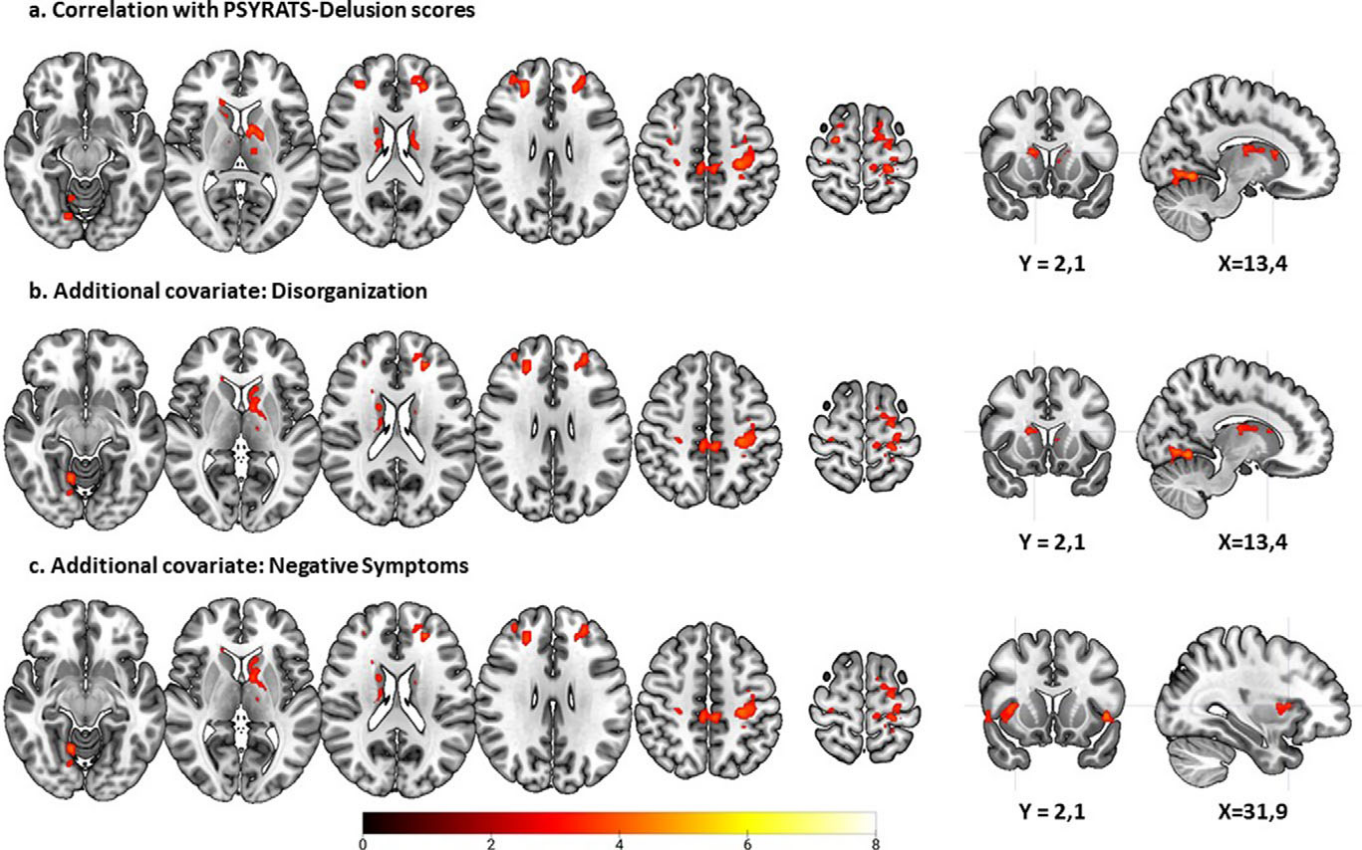
RPE-associated activations in the schizophrenia patients inversely correlated with PSYRATS delusion scores. Images are displayed in radiological convention (left is right). The color bar depicts *z* values.

Brain activations in the schizophrenia group showed an inverse correlation with PSYRATS delusion scores – , that is, patients with higher scores showed lower activation – in clusters in the caudate nucleus and thalamus bilaterally and in the left pallidum (left hemisphere: cluster size (voxels) = 587, *z* = 4.33, *p* < 0.001, MNI coordinates (*x*, *y*, *z*): −10, 4, 6; right hemisphere: cluster size (voxels)=107, *z* = 4.01, *p* = 0.02, MNI coordinates (*x*, *y*, *z*): 10, 16, 14). Inverse correlations were also seen in frontal regions with two clusters on the left [cluster size (voxels) = 1465, *z* = 4.38, *p* < 0.001, MNI coordinates (*x*, *y*, *z*): −30, −28, 48; cluster size (voxels) = 188, *z* = 4.74, *p* < 0.001, MNI coordinates (*x*, *y*, *z*): −26, 40, 22)], and two clusters on the right that involved the superior and middle frontal, precentral and postcentral gyri bilaterally [cluster size (voxels) = 525, *z* = 5.18, *p* < 0.001, MNI coordinates (*x*, *y*, *z*): 22, 38, 36), and cluster size (voxels)=171, *z* = 4.21, *p* = 0.001, MNI coordinates (*x*, *y*, *z*): 22, −6, 66)]. In the left hemisphere, inverse RPE-associated activation also extended to the supplementary motor area, the middle cingulate gyrus, the inferior and superior parietal cortex, the supramarginal gyrus, and the paracentral lobule. A further small cluster (cluster size (voxels) = 118, *z* = 4.23, *p* = 0.01, MNI coordinates (*x*, *y*, *z*): −62, −26, 0) was seen in the left middle temporal gyrus (Figure 5a; Supplementary Table S2). No significant clusters of positive association with RPE were observed.

In order to examine the possible influence of other symptoms on the above findings, the analysis was repeated including PANSS disorganization scores as a covariate. After doing this, most of the above clusters of significant inverse correlation remained, although those in the right precentral gyrus and left middle temporal gyrus were no longer seen (Figure 5b). When PANSS negative symptom scores were instead entered as a covariate, one of the two clusters in the right frontal cortex disappeared, as did the clusters in the right postcentral gyrus, the right middle cingulate gyrus and thalamus, and the left middle temporal gyrus (see Figure 5c and Supplementary Table S3).

We also examined the effect of controlling for antipsychotic dose (in chlorpromazine equivalents) on the findings. When this was added as a covariate, a similar pattern of clusters of inverse correlation with delusion scores continued to be found, but both the subcortical and cortical clusters became larger, with the latter now extending to parietal and occipital regions bilaterally (Supplementary Figure S2a and Table S6).

### RPE-associated activations in relation to IRIS scores

**Figure 6.**
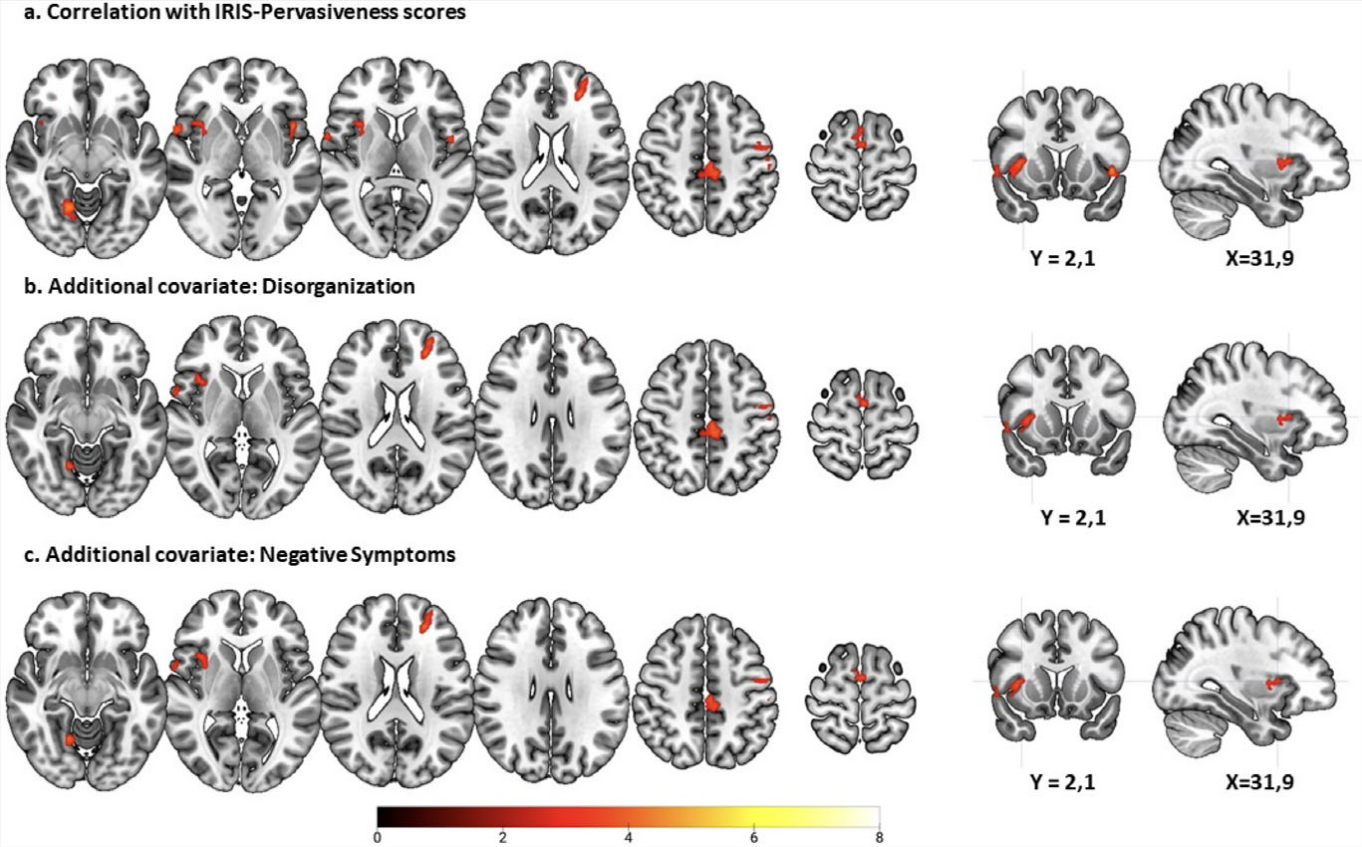
RPE-associated activations in the schizophrenia patients inversely correlated with IRIS pervasiveness scores. Images are displayed in radiological convention (left is right). The color bar depicts *z* values.

Brain activations in the schizophrenia patients showed an inverse correlation with IRIS pervasiveness scores in four main clusters. One of these was in the right putamen (*n* = 146 voxels, *z* = 3.95, *p* = 0.005, MNI coordinates (*x*, *y*, *z*): 32, 6, 6). Three cortical clusters were seen in the left middle and superior frontal gyrus and bilaterally in the supplementary motor area, middle cingulate gyrus, the insula, and the Rolandic operculum (*n* = 94 voxels, *z* = 3.85, *p* = 0.04, MNI coordinates (*x*, *y*, *z*): 0, −8, 72; and *n* = 193 voxels, *z* = 4.19, *p* < 0.001, MNI coordinates (*x*, *y*, *z*): −4, −24, 46; and *n* = 152 voxels, *z* = 4.23, *p* = 0.004, MNI coordinates (*x*, *y*, *z*): 56, −6, 14). The left-sided clusters also extended to the precentral and postcentral gyrus and the inferior parietal lobe and the right-sided cluster extended to the temporal pole and visual cortex (Figure 6; Supplementary Table S4).

When disorganization was added as a covariate, all the above clusters remained apart from that in the left Rolandic operculum and the insula (see Figure 6b; see also Supplementary Table S5). When negative symptom scores were added as a covariate, the clusters again remained similar apart from those in the left insula and the right Rolandic operculum, which disappeared (Figure 6c; see also Supplementary Table S5). Adding antipsychotic dose in CPZ equivalents as a covariate had little effect on the findings (see Figure S2b and Table S6).

## Discussion

This study found that RPE signaling was associated with a pattern of widespread activation involving the basal ganglia, the orbitofrontal and the lateral prefrontal cortex, among other areas, in healthy controls. There were only minor differences from this pattern in patients with schizophrenia. However, within the patient group, scores for both delusions and referentiality were associated with clusters of reduced RPE-related activation, which involved the basal ganglia, though not specifically the ventral striatum, as well as the prefrontal cortex and other cortical regions.

The pattern of RPE-associated activations we found in the healthy controls involved, as expected, some regions classically associated with reward processing, including the basal ganglia (the caudate nucleus, including its ventral sector, and the putamen) and the lateral prefrontal cortex. Beyond this, we found activations in the medial superior frontal cortex, the posterior cingulate gyrus, the precentral gyrus, the bilateral inferior parietal cortex extending to visual areas, and the hippocampus bilaterally. Many of these regions were also found in a meta-analysis of brain activations associated with prediction errors by Corlett et al. (2022), including analyses for secondary reward (monetary or non-monetary), appetitive reward and any reward (i.e., both primary and secondary). The major exception was the hippocampus, which was not activated in any of Corlett et al.’s (2022) meta-analyses, although they found activation of the parahippocampal gyrus in several of them.

As described in the introduction, brain functional changes during reward anticipation and reward feedback/delivery are well-established in schizophrenia. However, the results concerning RPE are currently conflicting, with one meta-analysis (Yaple et al., 2021) finding no differences between patients and healthy controls and another (Yang et al., 2024) finding changes in the direction of both increased and decreased activation in different brain regions. Our finding of only two small clusters of difference in RPE-associated activations between patients with schizophrenia and controls, both located in the occipital cortex, an area that is not implicated in reward processing at the theoretical level, tend to align with the former negative finding, and suggests that if there are changes in RPE signaling in schizophrenia as a whole, they may ultimately turn out to be subtle.

It is of course still possible that RPE signaling is altered in association with particular symptoms within schizophrenia. Our study provides support for this view, finding regions of significant inverse correlation with delusion scores in the basal ganglia and the lateral frontal cortex, as well as in the thalamus, the pre-and post-central gyrus, the supplementary motor area, and parts of the temporal and parietal cortex. There was a not-dissimilar pattern of correlation with scores on a referentiality scale. As far as we know, no other studies have examined RPE in relation to delusions. However, as noted in the introduction, a few studies have examined its association with positive symptoms, where the findings have been mixed. Gradin et al. (2011) found that increasing severity of psychotic symptoms was associated with decreased RPE-related activations to water reward in the right insula, the right amygdala–hippocampal complex and the midbrain; however, it should be noted that these authors’ measure of psychotic symptoms appeared to be based on summed positive and negative symptom scores. In contrast, another study found no association with psychotic symptoms in a mid-brain ROI in a study using monetary reward (Murray et al., 2008), and two more studies had negative or equivocal findings for positive symptoms in the ventral striatum (Culbreth et al., 2016; Katthagen et al., 2020).

Our finding of an inverse association between RPE-associated activations and delusions/referentiality is on the face of it opposite to what would be predicted on the basis of the aberrant salience theory, which argues that these symptoms are a consequence of pathologically increased RPE signaling caused by a functional dopamine excess. However, there may be ways of resolving this apparent contradiction. Firstly, Juckel et al. (2006) have argued that increased and chaotic dopamine activity in schizophrenia could act to increase ‘noise’ in the reward system, which might then drown out or otherwise interfere with RPE signaling to stimuli genuinely associated with reward. Secondly, Heinz and Schlagenhauf (2010) have pointed to differences in timescale that exist between the phasic firing of dopamine neurons that gives rise to RPE signaling (which is measured in milliseconds) and the measurement of brain functional changes during fMRI (which occurs over several seconds). This might mean that changes detected in the latter might not directly reflect changes in the former, but instead the response of a dysfunctional reward network. They found a degree of support for this argument in a study by Knutson et al. (2004) in which eight healthy subjects performed a monetary reward task after receiving amphetamine or placebo. Task performance on amphetamine (which would have increased dopaminergic activity) was associated with a less extensive pattern of reward anticipation-associated activation than on placebo. Further examination of ROIs in the ventral striatum and medial frontal cortex revealed a complex pattern of drug-related increases and decreases in activation at different levels of monetary reward.

In conclusion, we found evidence that both delusions and referential ideation in schizophrenia are associated with changes in RPE signaling, as indexed by brain activations during a probabilistic monetary reward task. These changes were seen in the context of only minor differences between patients and controls, and were in the opposite direction to that predicted by the aberrant salience theory, that is, the symptoms were associated with reduced rather than increased RPE-related activation. Some limitations need to be acknowledged. Thus, while additional analyses suggested that the findings were not an artifact of presence of negative or disorganization symptoms in the patients, to fully deal with this potential confound it would be necessary to compare groups of patients with and without delusions that were matched for presence of other classes of symptom. Also, our patient group showed a moderate symptom severity, which could have limited the power of correlational analysis to detect changes associated with delusions/referentiality. A more detailed characterization of delusional symptoms would be also helpful to study the influence of different types of delusions, for example, persecutory versus non-persecutory. A final limitation is that all the patients in the study were taking antipsychotic medication: however, while this may have influenced the findings in the comparison between patients and healthy controls, it is less likely that it would have played a role in the findings with respect to delusions and referentiality.

## Supporting information

García-León et al. supplementary material García-León et al. supplementary material

## Supplementary material

The supplementary material for this article can be found at http://doi.org/10.1017/S0033291724003258.
