# Supplementary material for: Are delusions and/or referentiality associated with aberrant reward prediction error (RPE) signaling? Evidence from fMRI using a probabilistic monetary reward task
Source: Psychol Med. 2025 Feb 20;55:e55. doi: 10.1017/S0033291724003258 (PMC12080658; doi:10.1017/S0033291724003258)
Supplement: García-León et al. supplementary material [file S0033291724003258sup001.docx]

**Supplementary Material for: Reward prediction error and its relationship to delusions and referentiality in schizophrenia: examination using fMRI and a probabilistic reward learning task. García-León et al.**

**Supplementary Results**

**Table S1**. Co-ordinates for clusters of RPE-associated activations in healthy controls, schizophrenia patients and controls and for significant differences between groups.

|  |  | **MNI Coordinates** | | |  |  |  |
| --- | --- | --- | --- | --- | --- | --- | --- |
| **Region** | **Hemisphere** | **x** | **y** | **z** | **Z-value** | ***k*** | ***p*** |
| ***Patients*** |  |  |  |  |  |  |  |
| **Inferior occipital gyrus** | **L** | **-36** | **-84** | **-10** | **12.3** | **75127** | **<.001** |
|  | R | 32 | -86 | -12 | 11.5 |  |  |
| Middle occipital gyrus | R | 34 | -90 | 2 | 11.7 |  |  |
|  | L | -30 | -90 | -2 | 11.6 |  |  |
| Lingual gyrus | R | 22 | -88 | -6 | 11.1 |  |  |
|  | L | -26 | -88 | -12 | 11 |  |  |
| Fusiform gyrus | L | -34 | -52 | -20 | 10.4 |  |  |
|  | R | 36 | -46 | -20 | 10.2 |  |  |
| Calcarine cortex | R | 16 | -96 | -4 | 10.3 |  |  |
| Inferior parietal cortex | L | -46 | -48 | 50 | 9.18 |  |  |
|  | R | 42 | -54 | 48 | 6.13 |  |  |
| Inferior temporal gyrus | L | -50 | -50 | -16 | 8.71 |  |  |
|  | R | 56 | -44 | -10 | 8.1 |  |  |
| Posterior cingulate gyrus | L | 2 | -38 | 30 | 8.62 |  |  |
| Cerebellum | L | -4 | -74 | -20 | 8.4 |  |  |
|  | R | 26 | -64 | -32 | 7.55 |  |  |
| Inferior frontal gyrus (pars triangularis) | L | -46 | 36 | 10 | 8.37 |  |  |
| Vermis |  | 0 | -54 | -36 | 8.29 |  |  |
| Putamen | R | 18 | 12 | -8 | 7.99 |  |  |
|  | L | -14 | 12 | -8 | 7.97 |  |  |
| Superior parietal cortex | L | -30 | -64 | 48 | 7.96 |  |  |
|  | R | 22 | -66 | 56 | 6.3 |  |  |
| Middle cingulate gyrus |  | 0 | -28 | 36 | 7.86 |  |  |
|  | R | 6 | 2 | 30 | 6.3 |  |  |
|  | L | -2 | -10 | 36 | 5.72 |  |  |
| Precentral | L | -42 | 2 | 30 | 7.73 |  |  |
| Amygdala | R | 24 | 0 | -16 | 7.6 |  |  |
| Hippocampus | L | -18 | -2 | -20 | 6.97 |  |  |
|  | R | 16 | -32 | -2 | 6.37 |  |  |
| Angular gyrus | L | -38 | -62 | 46 | 6.84 |  |  |
|  | R | 24 | -60 | 48 | 6.37 |  |  |
| Anterior cingulate gyrus | L | -4 | 10 | 26 | 6.84 |  |  |
| Middle temporal gyrus | L | -54 | -38 | -12 | 6.72 |  |  |
| Precuneus | L | -1 | -56 | 68 | 6.71 |  |  |
|  | R | 4 | -48 | 14 | 5.71 |  |  |
| Superior frontal gyrus | L | -20 | 16 | 58 | 6.43 |  |  |
| Inferior frontal gyrus (pars orbitalis) | L | -50 | 22 | -4 | 6.33 |  |  |
|  | R | 30 | 42 | -10 | 5.71 |  |  |
| Caudate | R | 16 | 4 | 14 | 6.33 |  |  |
| Thalamus | L | -8 | -14 | 8 | 6.23 |  |  |
|  | R | 8 | -4 | 10 | 5.91 |  |  |
| Middle frontal gyrus (pars orbitalis) | L | -40 | 48 | -4 | 6.17 |  |  |
| Paracentral gyrus | R | 4 | -42 | 74 | 6.12 |  |  |
| Precentral | L | -48 | 8 | 16 | 6.03 |  |  |
| Middle frontal gyrus | L | -36 | 50 | 6 | 6.01 |  |  |
|  | R | 48 | 40 | 22 | 5.82 |  |  |
| Calcarine | L | -12 | -70 | 8 | 5.88 |  |  |
| Inferior frontal gyrus (pars opercularis) | L | -50 | 10 | 28 | 5.84 |  |  |
| **Supplementary motor area** | R | **4** | **14** | **68** | **4.81** | **101** | **0.03** |
|  | L | -4 | -2 | 70 | 4.08 |  |  |
| ***Controls*** |  |  |  |  |  |  |  |
| **Middle occipital cortex** | **R** | **28** | **-90** | **8** | **11.5** | **32070** | **<.001** |
|  | L | -24 | -64 | 36 | 7.37 |  |  |
| Inferior occipital cortex | L | -36 | -84 | -10 | 11.2 |  |  |
|  | R | 32 | -86 | -12 | 10.6 |  |  |
| Fusiform gyrus | R | 36 | -70 | -12 | 9.62 |  |  |
|  | L | -36 | -54 | -20 | 9.04 |  |  |
| Lingual gyrus | R | 20 | -90 | -10 | 9.49 |  |  |
| Cerebellum | L | -34 | -76 | -22 | 8.33 |  |  |
|  | R | 8 | -78 | -18 | 7 |  |  |
| Inferior temporal gyrus | L | -44 | -64 | -6 | 8.26 |  |  |
| Inferior parietal cortex | L | -48 | -44 | 48 | 7.64 |  |  |
| Vermis |  | 0 | -58 | -36 | 6.74 |  |  |
| Superior parietal cortex | L | -20 | -60 | 46 | 6.62 |  |  |
| **Inferior frontal gyrus (pars triangularis)** | **L** | **-40** | **40** | **12** | **7.89** | **17441** | **<.001** |
| Putamen | L | -30 | -16 | -2 | 6.84 |  |  |
|  | R | 20 | 4 | 12 | 5.4 |  |  |
| Inferior frontal gyrus (pars orbitalis) | L | -52 | 22 | -2 | 6.75 |  |  |
| Middle frontal gyrus | L | -42 | 58 | -2 | 6.57 |  |  |
| Olfactory | R | 12 | 14 | -14 | 6.5 |  |  |
|  | L | -12 | 12 | -14 | 6.05 |  |  |
| Precentral | L | -42 | 4 | 32 | 6.18 |  |  |
| Middle frontal gyrus | L | -10 | 38 | 48 | 6.15 |  |  |
| Thalamus | L | -24 | -30 | 2 | 5.72 |  |  |
| Middle frontal gyrus | L | -38 | 14 | 42 | 5.64 |  |  |
| Superior frontal gyrus | L | -22 | 14 | 58 | 5.41 |  |  |
| Parahippocampal gyrus | L | -18 | 32 | 50 | 5.36 |  |  |
| Caudate | L | -8 | 14 | -2 | 5.25 |  |  |
| **Inferior frontal gyrus (pars triangularis)** | **R** | **54** | **38** | **12** | **5.79** | **2235** | **<.001** |
| Inferior frontal gyrus (pars orbitalis) | R | 40 | 42 | -16 | 5.68 |  |  |
| Middle frontal gyrus | R | 40 | 28 | 42 | 5.38 |  |  |
| Inferior frontal gyrus (pars opercularis) | R | 48 | 8 | 24 | 4.77 |  |  |
| Precentral gyrus | R | 50 | 10 | 32 | 4.44 |  |  |
| **Posterior cingulate gyrus** | **L** | **-2** | **-34** | **32** | **6.57** | **1689** | **<.001** |
|  | R | 10 | -42 | 20 | 3.79 |  |  |
| Precuneus | R | 4 | -48 | 14 | 5.11 |  |  |
|  | L | 0 | -48 | 36 | 4.39 |  |  |
| Middle cingulate gyrus | R | 4 | -38 | 46 | 4.9 |  |  |
| Middle cingulate gyrus | **R** | **6** | **0** | **30** | **4.49** | **163** | **0.002** |
| Anterior cingulate gyrus | L | -4 | 8 | 28 | 4.23 |  |  |
| **Superior frontal gyrus** | **R** | **26** | **-6** | **48** | **4** | **138** | **0.006** |
| Middle Frontal gyrus | R | 34 | 14 | 60 | 3.94 |  |  |
| ***Controls>Patients*** |  |  |  |  |  |  |  |
| **Middle occipital cortex** | **L** | **-22** | **-88** | **6** | **4.36** | **186** | **0.001** |
| **Middle occipital cortex** | **R** | **28** | **-90** | **10** | **4.91** | **151** | **0.004** |

*Note:* Whole-brain voxel-wise statistical tests were carried out with a *p* < 0.05, cluster correction for multiple comparisons and a cluster-forming threshold of *z* > 3.1 (*p* < 0.001) using Gaussian Random Field correction.

**Table S2**. Co-ordinates for clusters of RPE-associated activations negatively correlated with PSYRATS-delusion scores in schizophrenia patients.

|  |  | **MNI Coordinates** | | |  |  |  |
| --- | --- | --- | --- | --- | --- | --- | --- |
| **Region** | **Hemisphere** | **x** | **y** | **z** | **Z-value** | ***k*** | ***p*** |
| **Postcentral gyrus** | **L** | **-30** | **-28** | **48** | **4.38** | **1465** | **<.001** |
| Middle cingulate gyrus | R | 6 | -32 | 48 | 4.08 |  |  |
|  | L | -6 | -30 | 50 | 3.94 |  |  |
| Paracentral gyrus | L | -12 | -28 | 74 | 4.07 |  |  |
| Superior frontal gyrus | L | -20 | -2 | 68 | 4.05 |  |  |
| Inferior parietal cortex | L | -46 | -24 | 40 | 3.91 |  |  |
| Precentral gyrus | L | -24 | -12 | 66 | 3.86 |  |  |
| Superior parietal cortex | L | -26 | -44 | 64 | 3.76 |  |  |
| **Caudate** | **L** | **-10** | **4** | **6** | **4.33** | **587** | **<.001** |
|  |  | 16 | 2 | 20 | 3.83 |  |  |
| Thalamus | L | -10 | -8 | 14 | 4.13 |  |  |
|  | R | 10 | -10 | 10 | 3.94 |  |  |
| **Superior frontal gyrus** | **R** | **22** | **38** | **36** | **5.18** | **525** | **<.001** |
| Middle frontal gyrus | R | 30 | 38 | 44 | 4.21 |  |  |
| **Lingual gyrus** | **R** | **14** | **-60** | **-8** | **4.72** | **223** | **<.001** |
| **Middle frontal gyrus** | **L** | **-26** | **40** | **22** | **4.74** | **188** | **0.001** |
| **Superior frontal gyrus** | **R** | **22** | **-6** | **66** | **4.21** | **171** | **0.001** |
| Precentral gyrus | R | 30 | -12 | 56 | 3.94 |  |  |
| Middle frontal gyrus | R | 20 | 6 | 66 | 3.66 |  |  |
| **Middle temporal gyrus** | **L** | **-62** | **-26** | **0** | **4.23** | **118** | **0.01** |
| **Postcentral gyrus** | **R** | **26** | **-26** | **50** | **4.42** | **116** | **0.02** |
| Precentral gyrus | R | 32 | -26 | 58 | 3.81 |  |  |
| Paracentral gyrus | R | 18 | -30 | 56 | 3.2 |  |  |
| **Caudate** | **R** | **10** | **16** | **14** | **4.01** | **107** | **0.02** |
| **Cerebellum** | **R** | **26** | **-52** | **-28** | **3.9** | **97** | **0.04** |

*Note:* Whole-brain voxel-wise statistical tests were carried out with a *p* < 0.05, cluster correction for multiple comparisons and a cluster-forming threshold of *z* > 3.1 (*p* < 0.001) using Gaussian Random Field correction.

**Table S3**. Co-ordinates for clusters of RPE-associated activations negatively correlated with PSYRATS-delusion scores in schizophrenia patients with disorganization and negative symptoms as covariates.

|  |  | **MNI Coordinates** | | |  |  |  |
| --- | --- | --- | --- | --- | --- | --- | --- |
| **Region** | **Hemisphere** | **x** | **y** | **z** | **Z-value** | **k** | **p** |
| *Additional covariate: disorganization* | | | | | | | |
| **Postcentral gyrus** | **L** | **-28** | **-26** | **48** | **4.37** | **1151** | **<.001** |
| Middle cingulate gyrus | L | -4 | -32 | 50 | 4.3 |  |  |
|  | R | 6 | -32 | 48 | 4.16 |  |  |
| Precentral gyrus | L | -12 | -28 | 74 | 4.2 |  |  |
| Paracentral lobule | L | -20 | -28 | 54 | 4.09 |  |  |
| Superior frontal gyrus | L | -16 | -6 | 64 | 3.99 |  |  |
| Superior parietal cortex | L | -18 | -46 | 62 | 3.48 |  |  |
| Inferior parietal cortex | L | -34 | -42 | 54 | 3.46 |  |  |
| Supramarginal gyrus | L | -30 | -40 | 44 | 3.36 |  |  |
| Supplementary motor area | L | -12 | 2 | 64 | 3.3 |  |  |
| **Thalamus** | **L** | **-10** | **-8** | **14** | **4.14** | **484** | **<.001** |
|  | R | 10 | -10 | 12 | 3.84 |  |  |
| Caudate | L | -10 | 4 | 8 | 4.03 |  |  |
|  | R | 16 | 2 | 20 | 3.84 |  |  |
| Pallidum | L | -18 | -2 | 6 | 3.61 |  |  |
| **Middle frontal gyrus** | **R** | **24** | **38** | **30** | **4.5** | **295** | **<.001** |
| **Lingual gyrus** | **R** | **14** | **-60** | **-8** | **4.63** | **197** | **<.001** |
| Cerebellum | R | 16 | -74 | -12 | 3.59 |  |  |
| **Superior frontal gyrus** | **L** | **-26** | **40** | **22** | **4.67** | **180** | **0.001** |
| **Postcentral gyrus** | **R** | **26** | **-26** | **50** | **4.29** | **106** | **0.02** |
| Precentral gyrus | R | 30 | -26 | 60 | 4.01 |  |  |
| **Caudate** | **R** | **16** | **26** | **8** | **3.89** | **99** | **0.03** |
| *Additional covariate: negative symptoms* | |  |  |  |  |  |  |
| **Precentral gyrus** | **L** | **-24** | **-30** | **68** | **4.35** | **594** | **<.001** |
| Postcentral gyrus | L | -26 | -36 | 60 | 4.18 |  |  |
| Supplementary motor area | L | -20 | -28 | 54 | 4.14 |  |  |
| Middle cingulate gyrus | L | -6 | -30 | 50 | 3.85 |  |  |
| **Superior Frontal gyrus** | R | **22** | **38** | **36** | **4.78** | **373** | **<.001** |
| Middle frontal gyrus | R | 30 | 38 | 44 | 3.9 |  |  |
| **Supplementary motor area** | L | **-14** | **-4** | **64** | **4.26** | **189** | **<.001** |
| **Thalamus** | L | **-10** | **-8** | **14** | **4.06** | **159** | **0.003** |
| Caudate | L | -10 | 4 | 6 | 3.9 |  |  |
| Pallidum | L | -18 | -2 | 8 | 3.81 |  |  |
| **Middle frontal gyrus** | L | **-26** | **40** | **22** | **4.74** | **131** | **0.009** |
| Superior frontal gyrus | L | -22 | 48 | 22 | 3.82 |  |  |
| **Caudate** | R | **16** | **26** | **8** | **4.24** | **131** | **0.009** |
| **Lingual gyrus** | R | **12** | **-60** | **-8** | **4.48** | **124** | **0.01** |
| Cerebellum | R | 10 | -70 | -6 | 4.15 |  |  |

*Note:* Whole-brain voxel-wise statistical tests were carried out with a *p* < 0.05, cluster correction for multiple comparisons and a cluster-forming threshold of *z* > 3.1 (*p* < 0.001) using Gaussian Random Field correction.

**Table S4**. Co-ordinates for clusters of RPE-associated activations negatively correlated IRIS-pervasiveness scores in schizophrenia patients.

|  |  | **MNI Coordinates** | | |  |  |  |
| --- | --- | --- | --- | --- | --- | --- | --- |
| **Region** | **Hemisphere** | **x** | **y** | **z** | **Z-value** | ***k*** | ***p*** |
| **Postcentral gyrus** | **L** | **-52** | **-6** | **40** | **4.59** | **295** | **<.001** |
| Precentral gyrus | L | -46 | -2 | 48 | 3.34 |  |  |
| Supramarginal gyrus | L | -52 | -26 | 32 | 3.21 |  |  |
| Inferior parietal cortex | L | -46 | -24 | 38 | 3.18 |  |  |
| **Middle cingulate gyrus** | **L** | **-4** | **-24** | **46** | **4.19** | **193** | **<.001** |
|  | R | 6 | -30 | 50 | 3.53 |  |  |
| **Rolandic operculum** | **R** | **56** | **-6** | **14** | **4.23** | **152** | **0.004** |
| Temporal pole | R | 60 | 8 | -4 | 4.2 |  |  |
| **Insula** | **R** | **36** | **14** | **4** | **3.95** | **146** | **0.005** |
| Putamen | R | 32 | 6 | 6 | 3.63 |  |  |
| **Lingual gyrus** | **R** | **16** | **-60** | **-8** | **4.84** | **145** | **0.005** |
| **Middle frontal gyrus** | **L** | **-26** | **42** | **22** | **4.63** | **110** | **0.02** |
| **Temporal pole** | **L** | **-46** | **14** | **-2** | **4.63** | **100** | **0.03** |
| Insula | L | -44 | 4 | 0 | 3.88 |  |  |
| Rolandic operculum | L | -48 | 0 | 8 | 3.82 |  |  |
| **Supplementary motor area** | **L** | **0** | **-8** | **72** | **3.85** | **94** | **0.04** |
|  | R | 6 | 2 | 66 | 3.43 |  |  |

*Note:* Whole-brain voxel-wise statistical tests were carried out with a *p* < 0.05, cluster correction for multiple comparisons and a cluster-forming threshold of *z* > 3.1 (*p* < 0.001) using Gaussian Random Field correction.

**Table S5.** Co-ordinates for clusters of RPE-associated activations negatively correlated with IRIS-pervasiveness scores in schizophrenia patients including disorganization and negative symptoms as covariate.

|  |  | **MNI Coordinates** | | |  |  |  |
| --- | --- | --- | --- | --- | --- | --- | --- |
| **Region** | **Hemisphere** | **x** | **y** | **z** | **Z-value** | **k** | ***p*** |
| *Additional covariate: disorganization* | | | | | | | |
| **Postcentral gyrus** | **L** | **-52** | **-6** | **40** | **4.33** | **222** | **<.001** |
| Precentral gyrus | L | -36 | -4 | 44 | 3.56 |  |  |
| **Middle cingulate gyrus** | **L** | **-6** | **-30** | **50** | **4.31** | **210** | **<.001** |
|  | R | 6 | -30 | 50 | 3.44 |  |  |
| Supplementary motor area | L | -8 | -18 | 54 | 3.69 |  |  |
| **Supplementary motor area** | **L** | **-2** | **-4** | **66** | **4** | **134** | **0.008** |
|  | R | 12 | 2 | 56 | 3.63 |  |  |
| **Temporal pole superior** | **R** | **58** | **10** | **0** | **4.25** | **103** | **0.03** |
| **Insula** | **R** | **38** | **16** | **2** | **4.2** | **101** | **0.03** |
| **Superior frontal gyrus** | **L** | **-26** | **42** | **22** | **4.44** | **100** | **0.03** |
| Middle frontal gyrus | L | -28 | 50 | 20 | 3.99 |  |  |
| **Lingual gyrus** | **R** | **16** | **-60** | **-8** | **4.48** | **94** | **0.04** |
| *Additional covariate: negative symptoms* | |  |  |  |  |  |  |
| **Precentral gyrus** | **L** | **-52** | **-6** | **40** | **4.55** | **246** | **<.001** |
| Postcentral gyrus | L | -46 | -12 | 40 | 4.46 |  |  |
| **Middle cingulate gyrus** | **L** | **-4** | **-24** | **46** | **4.11** | **144** | **0.005** |
| **Insula** | **R** | **38** | **16** | **2** | **3.82** | **113** | **0.02** |
| **Supplementary motor area** | **L** | **-2** | **-4** | **66** | **3.87** | **106** | **0.02** |
|  | R | 6 | 2 | 66 | 3.34 |  |  |
| **Temporal pole superior** | **R** | **58** | **10** | **-2** | **3.99** | **103** | **0.03** |
| **Superior frontal gyrus** | **L** | **-26** | **42** | **22** | **4.59** | **99** | **0.03** |
| Middle frontal gyrus | L | -28 | 50 | 20 | 3.74 |  |  |
| **Lingual gyrus** | **R** | **16** | **-60** | **-8** | **4.68** | **98** | **0.04** |

*Note:* Whole-brain voxel-wise statistical tests were carried out with a *p* < 0.05, cluster correction for multiple comparisons and a cluster-forming threshold of *z* > 3.1 (*p* < 0.001) using Gaussian Random Field correction.

**Parameter recoverability simulation analysis.**

We assessed parameter recoverability using simulated data with 500 iterations. Data were generated for a range of alpha and beta values between 0 and 1, based on the empirical data obtained in the task, and parameter values were estimated using the simulated data. True and estimated parameter values were significantly correlated (alpha r = 0.58, beta r = 0.38, both p < 0.001). Figure S1 shows the association between true and estimated alpha and beta values.

*
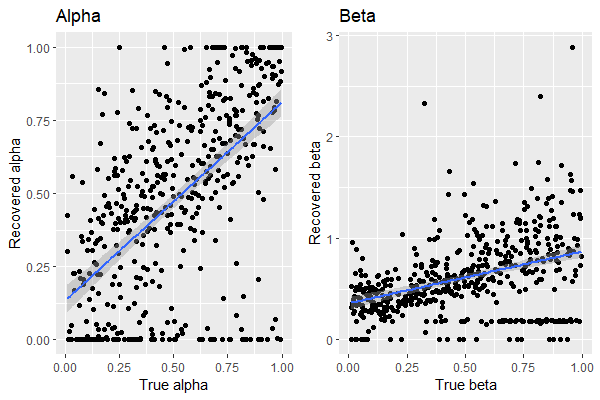
*

**Figure S1**. Association between true and estimated alpha and beta values.

**RPE-associated activations in relation to PSYRATS delusion scores and IRIS Pervasiveness scores with CPZ equivalents as covariate**

When CPZ equivalents is added as covariate, brain activations in the schizophrenia group showed an inverse correlation with PSYRATS delusion scores – ie patients with higher scores showed lower activation – in clusters similar to that found in the original analysis. Subcortically inverse correlations were found in the caudate, thalamus, pallidum and putamen bilaterally. Inverse correlations were also seen in frontal regions in the superior and middle frontal, precentral and postcentral gyri bilaterally. In the left hemisphere inverse RPE-associated activation also extended to the supplementary motor area, the middle cingulate gyrus, the inferior and superior parietal cortex, the supramarginal gyrus and the paracentral lobule. In the right hemisphere clusters extended to precentral and postcentral gyrus and parietal superior gyrus (see Figure S2 and Table Sx).

Analysis of correlation with IRIS Pervasiveness scores with CPZ equivalents as covariate, also show similar results to that found in the original analysis. Inverse correlations were found in the right putamen. Cortical clusters were seen in the left middle and superior frontal gyrus and bilaterally in the supplementary motor area, middle cingulate gyrus, the insula and the Rolandic operculum. The left-sided clusters also extended to the precentral and postcentral gyrus and the inferior parietal lobe and the right-sided cluster extended to the temporal pole and visual cortex

No significant clusters of positive association with RPE were observed.


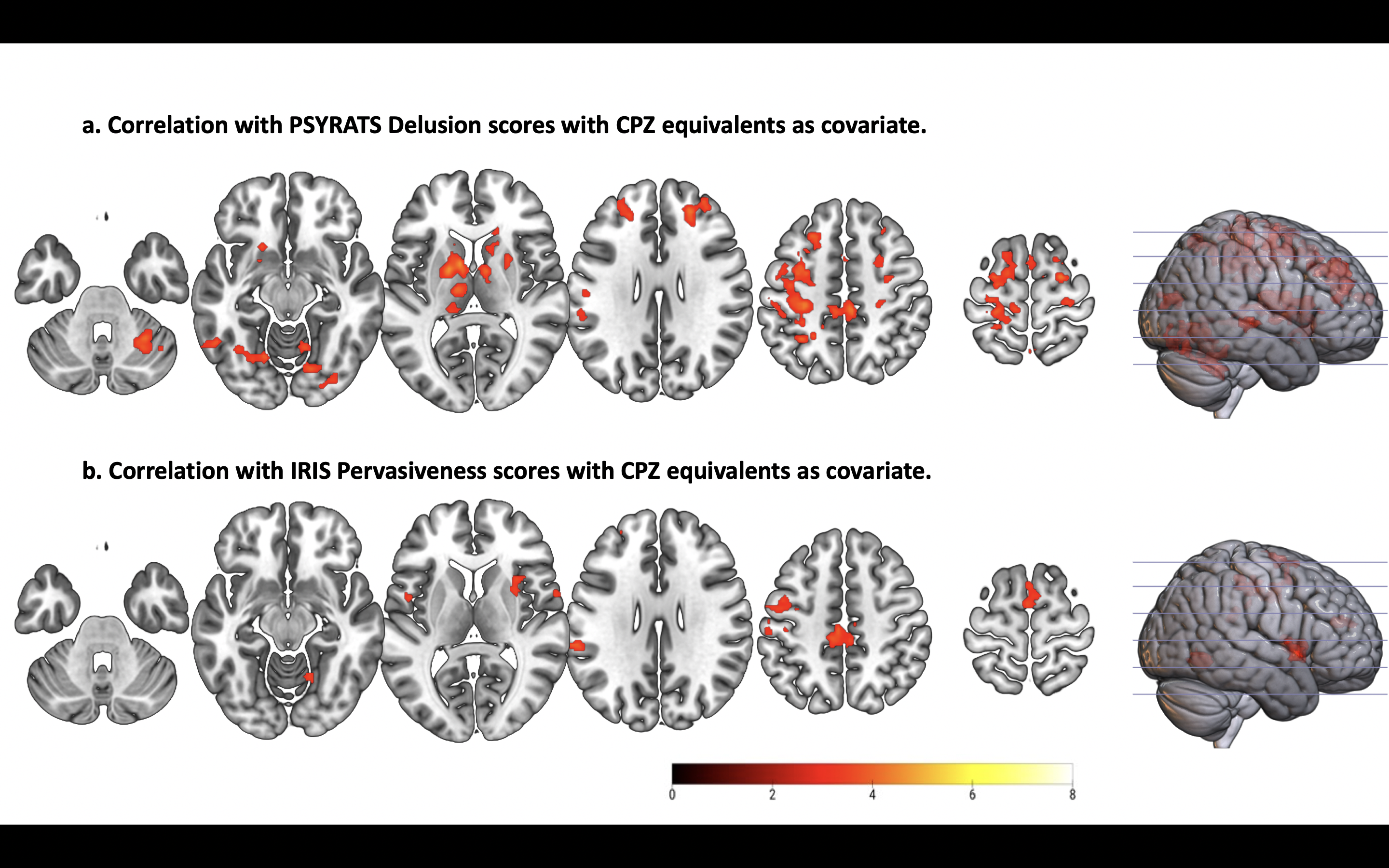


**Figure S2.** RPE-associated activations in the patients inversely correlated with PSYRATS delusion scores (a) and IRIS Pervasiveness scores (b) with CPZ equivalents as covariate. Images are displayed in radiological convention (left is right). Colour bar depicts *z* values.

**Table S6.** Co-ordinates for main peaks of clusters of RPE-associated activations negatively correlated with PSYRATS Delusion scores and IRIS-pervasiveness scores in schizophrenia patients including CPZ equivalents as covariate.

|  |  | **MNI Coordinates** | | |  |  |  |
| --- | --- | --- | --- | --- | --- | --- | --- |
| **Region** | **Hemisphere** | **x** | **y** | **z** | **Z-value** | **k** | ***p*** |
| *Correlation with PSYRATS Delusion scores* | | | | | | | |
| Precentral | L | -36 | -4 | 46 | 4.95 | 2846 | <.001 |
| Caudate | L | -10 | 4 | 6 | 4.82 | 1518 | <.001 |
| Lingual gyrus | R | 14 | -60 | -8 | 4.73 | 806 | <.001 |
| Superior frontal gyrus | R | 22 | 38 | 36 | 5.26 | 713 | <.001 |
| Superior frontal gyrus | R | 22 | -6 | 66 | 4.39 | 300 | <.001 |
| Lingual gyrus | L | -20 | -64 | -6 | 4.04 | 271 | <.001 |
| Inferior occipital cortex | R | 34 | -82 | -14 | 4.15 | 228 | <.001 |
| Postcentral | R | 26 | -26 | 50 | 4.42 | 203 | <.001 |
| Middle temporal gyrus | L | -66 | -26 | -2 | 4.28 | 201 | <.001 |
| Middle frontal gyrus | L | -26 | 40 | 22 | 4.65 | 190 | 0.001 |
| Supplementary Motor Area | R | 4 | 0 | 70 | 4.17 | 182 | 0.002 |
| Precuneus | L | -2 | -66 | 62 | 4.67 | 152 | 0.005 |
| Inferior temporal gyrus | L | -52 | -54 | -22 | 4.57 | 151 | 0.005 |
| Middle occipital cortex | L | -24 | -80 | 14 | 4.06 | 119 | 0.017 |
| Middle occipital cortex | R | 30 | -76 | 16 | 3.84 | 119 | 0.017 |
| Inferior parietal cortex | L | -30 | -52 | 50 | 4.54 | 112 | 0.023 |
| Middle frontal gyrus | L | -32 | 56 | 18 | 4.33 | 98 | 0.042 |
| Middle temporal gyrus | R | 64 | -26 | 0 | 4.34 | 94 | 0.049 |
| *Correlation with IRIS Pervasiveness scores* | | | | | | |  |
| Postcentral | L | -50 | -6 | 40 | 4.81 | 439 | <.001 |
| Middle cingulate gyrus | L | -4 | -24 | 46 | 4.26 | 249 | <.001 |
| Lingual gyrus | R | 16 | -60 | -8 | 4.87 | 220 | <.001 |
| Supplementary Motor Area | L | 0 | 10 | 64 | 3.97 | 195 | 0.001 |
| Insula | R | 36 | 14 | 4 | 3.96 | 169 | 0.003 |
| Temporal Pole Superior | L | -46 | 14 | -2 | 4.68 | 120 | 0.018 |
| Temporal Pole Superior | R | 60 | 8 | -4 | 4.12 | 115 | 0.022 |
| Middle frontal gyrus | L | -26 | 42 | 22 | 4.59 | 107 | 0.030 |
| Supramarginal gyrus | L | -60 | -34 | 28 | 4.01 | 97 | 0.046 |

*Note:* Whole-brain voxel-wise statistical tests were carried out with a *p* < 0.05, cluster correction for multiple comparisons and a cluster-forming threshold of *z* > 3.1 (*p* < 0.001) using Gaussian Random Field correction.

**Scatter plots of the associations between activation levels and symptoms scores in each region with significant correlations.**

For illustrative purposes, Figures S3 and S4 show scatter plots of associations between brain activation levels and symptoms (delusion and self-referentiality) in each region that show significant correlations (see Results section).

**
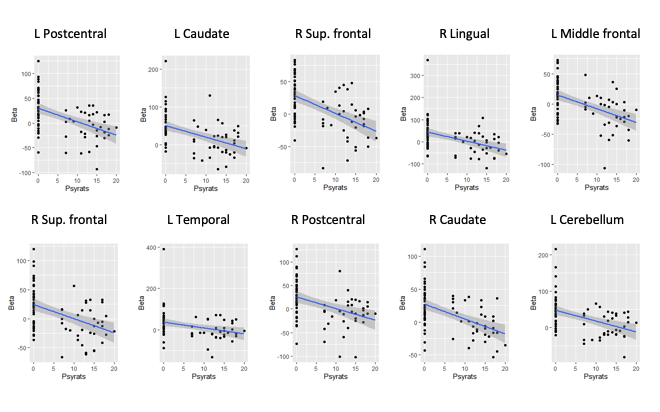
**

**Figure S3.** Scatterplots showing the associations between activation levels and Psyrats scores in each region with significant correlations.

**
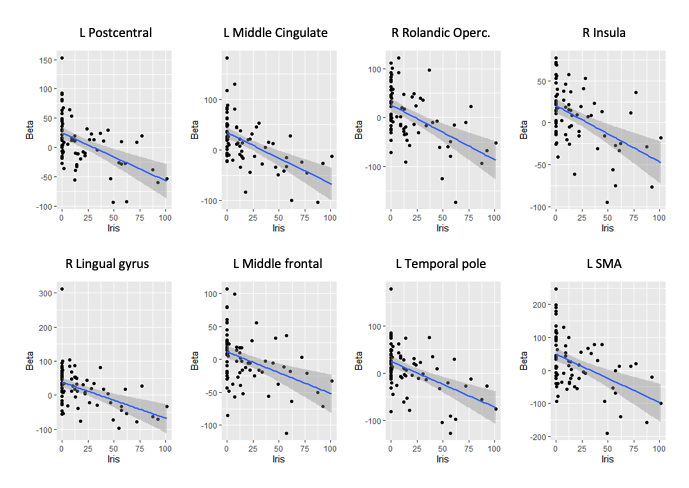
**

**Figure S4.** Scatterplots showing the associations between activation levels and IRIS scores in each region with significant correlations.
